# A narrative review of factors influencing rider performance and horse welfare in equestrian activities

**DOI:** 10.3389/fspor.2025.1744918

**Published:** 2026-01-22

**Authors:** Orsolya Balog, Krisztián Havanecz, Tamás Csányi, Csaba Ökrös, László Tóth, Tamás Berki

**Affiliations:** Hungarian University of Sports Science, Budapest, Hungary

**Keywords:** asymmetry, equestrian sports, fitness, horse welfare, rider biomechanics, training habits

## Abstract

Equestrian sport is a unique multi-species discipline in which the performance of a horse–rider dyad depends on the harmonious interaction of two athletes with distinct biomechanics and needs. Although the sport contributes substantially to the global economy and is the only Olympic event involving two species, research on rider-centered factors has been fragmented. Current narrative review centered peer-reviewed evidence addressing three questions: (RQ1) how rider biomechanics and posture influence horse performance and welfare; (RQ2) what causes and consequences rider asymmetry has; and (RQ3) how riders’ fitness, health and training practices affect performance and well-being. Electronic searches of five databases—namely PubMed, Web of Science, Scopus, SPORTDiscus and Google Scholar—covering 2000 to July 2024 retrieved 83 records; 17 studies met the inclusion criteria. Correct pelvic orientation, dynamic trunk control and symmetrical weight distribution were consistently associated with improved saddle pressure distribution and better equine gait. Asymmetries in riders’ posture, whether inherent or acquired, contributed to uneven loading and degraded performance, yet many riders were unaware of their imbalances. Studies on fitness and training showed that equestrians often neglect structured off-horse conditioning despite moderate-to-vigorous cardiovascular demands during riding. Targeted core training programmes, cross-training and nutritional support improved balance and reduced back pain. Taken together, the literature points to a need for holistic interventions that combine biomechanical assessment, correction of asymmetry and structured fitness programmes to support both the rider and their horse.

## 1 Introduction

Equestrian sports play a significant role in the global economy by driving investments in horse breeding, events, and training, which provide substantial economic benefits and support millions of jobs worldwide (1). Animal welfare is a key priority within equestrian sports, as welfare concerns have become a focal point for regulatory bodies. The Fédération Equestre Internationale (FEI) has responded with more rigorous welfare standards, emphasizing humane training practices, proper veterinary care, and ethical treatment of horses both in and out of competition (2).

Equestrian sports have a well-established place in the Olympic Games, however, they face challenges related to logistical and financial demands, as well as the costs associated with ensuring welfare standards. The International Olympic Committee (IOC) has been considering refining equestrian events to address these concerns, and the sport's future Olympic status remains a topic of ongoing discussion (3). Looking ahead, equestrian sports have the potential for growth in emerging markets, particularly in Asia and South America. The future success of equestrian sports will likely depend on addressing current challenges, enhancing accessibility, and adapting to modern economic and ethical expectations.

Equestrian sports involve a delicate relationship between riders and their equine partners, where factors such as rider biomechanics, posture, and fitness play critical roles in performance and horse welfare (4). Horse welfare refers to the comprehensive care and ethical treatment of horses, ensuring their physical and psychological well-being is prioritized over competitive or commercial interests. This includes suitable nutrition, safe stabling, humane training methods, and proper veterinary care. Additionally, horse welfare emphasizes the importance of safe competition environments, monitoring and minimizing risk of injury, and upholding humane treatment standards throughout the horse's athletic career and retirement. Ongoing education and research guide these efforts, striving to continuously improve practices that support the health, comfort, and safety of the horses (5).

Several studies have delved into the understanding how rider posture asymmetry, stirrup length, and other factors influence both rider comfort and the effectiveness in the saddle (4, 6). Additionally, rider asymmetry and its influence on equine locomotion have been explored by various researchers, underscoring the need for further understanding to address these issues for both rider performance and horse welfare (7–11).

These investigations highlight the complex nature of equestrian sports and emphasize the need for thorough research to improve both rider performance and horse well-being. Thus, this narrative review aims to consolidate findings from recent studies to provide a clear understanding of the intricate relationship between riders and their equine partners, based on equestrian sports expertise. Therefore, we address three key questions in this review 1) How do biomechanics and posture affect rider performance and horse welfare? 2) What role does asymmetry play in rider health and equine locomotion? 3) How can fitness and health improvements support optimal rider performance and welfare?

## 2 Material and methods

The literature search covered research from multiple databases, including studies published in English, across various design formats. Included studies span biomechanics, rider asymmetry, and training impact, with a focus on peer-reviewed equestrian sports literature from the past two decades. Studies were collected and critically reviewed by the first, second, and third authors. All the authors approved the final version.

This review used a narrative methodology given the heterogeneity of study designs, populations, and outcome measures in the literature (12). Following the narrative review approach, we formulated three research questions around rider biomechanics, asymmetry and fitness practices. The search strategy, selection criteria and analysis steps are described below.

### 2.1 Search strategy

An extensive literature search was conducted in July 2024. Five electronic databases—PubMed, Web of Science, Scopus, SPORTDiscus and Google Scholar—were searched using combinations of the terms “equestrian OR horse riding”, “rider biomechanics OR posture”, “rider asymmetry OR laterality”, “fitness OR physical training”, “injury OR pain”, and “equine welfare”. Manual searches of reference lists supplemented these sources. Searches were not limited by language at the outset, but only English-language articles were retained. Grey literature, conference abstracts and non-peer-reviewed materials were excluded. The search identified 83 records after duplicates were removed. Titles and abstracts were screened independently by two reviewers against the inclusion criteria: peer-reviewed empirical study; participants were riders of any equestrian discipline; study addressed at least one of the three research questions; and outcomes related to rider biomechanics, asymmetry, fitness/health or horse welfare. Full-text screening retained 17 studies for inclusion.

### 2.2 Data extraction and synthesis

For each included study we extracted: author(s) and year; country; design; sample characteristics; focus area; and key findings. Because the studies were heterogeneous in design and outcome measures, data were synthesised qualitatively. Studies were grouped into three categories corresponding to the research questions: rider biomechanics and posture (RQ1), rider asymmetry (RQ2) and rider fitness, health and training practices (RQ3). A table summarises the characteristics of the included studies. In the results section we first describe the overall characteristics and then present the findings for each research question. Numbers of studies per category are reported to provide transparency.

#### 2.2.1 Characteristics of the included studies

Supplementary Table S1 summarizes the 17 studies included in the review. The majority of these studies were observational, cross-sectional in design and were conducted in Europe or North America. In fact, over two-thirds of the studies took place in Europe (e.g., UK, Netherlands, Germany, etc.), with a smaller portion from North America (the USA and Canada) and only two originating from elsewhere (Korea and Australia). Sample sizes varied greatly from as few as five or six participants in the smallest studies to large survey-based studies with over 1,200 riders, reflecting a diversity of research approaches (including motion analysis, force measurements, surveys, and exercise interventions). This means the evidence base includes both laboratory-style biomechanical analyses and broad population surveys.

Of the 17 studies, seven addressed RQ1, five addressed RQ2 and five addressed RQ3 (focusing on rider biomechanics, rider asymmetry, and rider fitness/training, respectively). Notably, only two studies implemented a dedicated experimental intervention (one randomized trial and one quasi-experimental training program), while the remainder were observational studies. Dressage, show jumping, and general recreational riding were the most common disciplines represented among participants, although some studies included mixed or unspecified rider groups (one even focused on the tölt gait in Icelandic riding). Overall, the literature on rider-focused factors remains limited in scope and quality—most studies involved small convenience samples and cross-sectional designs—which underscores the need for further, more rigorous research in this area.

### 2.3 Rider biomechanics and posture

A total of seven studies examined rider biomechanics and posture (see Supplementary Table S1). Taken together, they show that correct pelvic orientation, dynamic trunk control and symmetrical weight distribution are more than aesthetic concerns: they directly influence saddle pressure patterns and equine gait quality. Clayton and Hobbs (13) found that riders who maintained a neutral pelvis and engaged their core generated lower peak pressures and aligned their center of mass (COM) more closely with that of the horse. Similarly, de Cocq et al. (8, 9) demonstrated that positioning the seat near the horse's COM reduced peak saddle forces. Conversely, heavier riders or those who shifted their weight forward increased forelimb loading and disrupted balance, underscoring the welfare implications of poor biomechanical efficiency. Although these studies highlight consistent trends, most were small cross-sectional designs; robust longitudinal trials are rare.

Hobbs et al. (14) delved deeper into postural asymmetry, strength and flexibility in riders. In a large cohort, high-level dressage riders with extensive experience displayed reduced lateral bending range of motion (ROM) to the left and asymmetric shoulder heights, suggesting greater musculature or possible pain on the right. Many experienced years of riding were linked to increased sitting pelvic asymmetry, which may contribute to restricted lateral bending in elite riders. Rather than promoting symmetry, the demands of advanced dressage may therefore exacerbate asymmetries and predispose riders to chronic back pain. These findings mirror the inherent asymmetries of horses themselves, which riders and trainers aim to correct; the horse's natural slant can influence the rider's position and may partly explain the ROM changes observed in research.

Subsequent work by Bye and Lewis (15) investigated whether footedness and handedness contribute to postural asymmetry. Their results indicate that these demands are unlikely to be a major factor; instead, riders compensate for asymmetry by relying on stirrups, often exhibiting pelvic obliquity and opposite shoulder tilt to maintain balance. While this strategy allows riders to remain centered despite pronounced postural deviations, it also highlights a broader issue: equestrians tend to prioritize the horse's preparation over their own. Warm-up sessions typically focusing on physical and mental preparation regarding the horse, not on loosening the rider. Over years of unilateral loading without sufficient conditioning or corrective exercises, asymmetries may worsen the posture and back pain becomes more prevalent. Collectively, these findings underline the need for exercise training protocols that support riders in achieving a straighter, more balanced and effective seat on the horse.

Geser-von Peinen et al. (16) examined the interaction between forces on the horse's back and rider movements. They showed that variability in rider posture and position leads to inconsistent force distributions, which can cause discomfort or strain for the horse. Wilkins et al. (17) further noted that coordination variability among competitive dressage riders influences the ability to maintain an “independent seat”, illustrating the fine motor control required for stability. Together these studies underscore the importance of balanced, controlled biomechanics in reducing strain on the horse's back and enhancing overall welfare and performance.

A separate line of inquiry addresses how stirrup length affects impact attenuation and rider comfort. Keener et al. (18) reported that supporting more weight through the legs with a shortened stirrup can reduce lumbar impacts and associated with injury risk. They emphasized that riders should be taught to engage lower-body and core strength to attenuate impacts effectively. Stapley et al. (19) compared different stirrup iron styles and found minimal effects on rider position or forces loaded on the horse's back, although some gait-specific differences emerged. Randle and Loy (20) noted that body proportions may influence optimal stirrup adjustments, highlighting the role of morphology. Bye and Lewis (21) compared saddle and stirrup forces during sitting trot, rising trot and sitting trot without stirrups on a simulator, finding significantly higher peak vertical forces when riding without stirrups. While training without stirrups can develop technique, it may also increase saddle forces. An often-overlooked factor is the material of riding equipment: leather saddles and stirrup straps stretch asymmetrically due to habits such as mounting from the left, which may exacerbate imbalances over time. These studies provide practical insights for optimizing stirrup-related variables to improve rider comfort and effectiveness.

In summary, the available evidence indicates that well-aligned posture, effective core engagement and appropriate stirrup use are fundamental to the rider's stability, horse comfort and performance (13). Nonetheless, much of the research relies on small, cross-sectional samples, and findings are sometimes conflicting. Larger, controlled studies are needed to confirm biomechanical guidelines and to evaluate interventions aimed at correcting asymmetries and enhancing rider conditioning.

### 2.4 Rider asymmetry—causes and effects

Five studies in Supplementary Table S1 (studies 4 and 8–11) focused explicitly on rider asymmetry and its effects on equine locomotion and welfare. Objective measurements consistently show left–right differences in shoulder height, pelvic tilt or stirrup force among riders. Yet survey data indicate that more than 60% of riders report “sidedness”, meanwhile the perceived asymmetry correlates moderately with measured inbalances (13). This disparity suggests that many riders do not fully recognize their own inbalances, underscoring the importance of objective evaluation (22).

Early work by Licka et al. (7) and de Cocq et al. (8, 9) identified structural asymmetries in riders' spines and pelvises and proposed that habitual riding patterns reinforce these imbalances. Symes and Ellis (10) and Roepsorff et al. (11) further highlighted that both congenital laterality and accumulated injuries contribute to asymmetry. Evidence from motor control research suggests that limb dominance influences postural control and coordination (23, 24). Additionally, Bussey (25) linked leg length discrepancies and pelvic tilt to shoulder rotation asymmetry in athletes. In the equestrian context, Eisersiö et al. (26) observed that highly experienced riders still struggle to perceive small rein-tension differences, indicating sensory overload during complex tasks. Collectively these findings imply that rider asymmetry arises from an interplay of inherent laterality, learned motor patterns and the challenges of processing multiple sensory inputs (27).

The consequences of asymmetry on equine locomotion are becoming clearer (28). Propulsive forces from the horse are transmitted to the rider, and uneven loading can exacerbate pelvic asymmetry (16). MacKechnie-Guire et al. (22) showed that inducing asymmetry in the rider increased the horse's lateral displacement and altered stride parameters. A survey and biomechanical analysis by the same group found that perceived sidedness corresponded moderately with measured asymmetry and that training for symmetrical load distribution improved objective measures (29). Studies on Icelandic tölters and show-jumping riders reported that asymmetry degraded gait quality and increased head movement, while also targeted stretching and physiotherapy improved symmetry and riding performance (6).

Recent work has deepened understanding of the mechanisms behind these effects. Hogg et al. (30) demonstrated that rider asymmetry and stress responses disrupt physiological synchronisation between horse and rider. Elbrønd and Schultz (31) described deep myofascial connections in the equine back and proposed how asymmetric rider loading might transmit strain through these chains, affecting both comfort and biomechanics. Gunst et al. (32) analysed data from 80 horse–rider pairs and found that when a rider collapsed on one hip, force increased on the opposite side of the saddle, whereas upper-body tilt increased force on the same side. Their multivariate analysis emphasised that asymmetric force distributions reflect complex interactions among horse morphology, saddle fit and rider posture; thus, all components must be considered when interpreting pressure data.

In addition to these empirical findings, practitioners note that horses themselves have congenital asymmetries, which riders and trainers also work to correct. The horse's natural slant influences the rider's position, which may contribute to the ROM changes observed in advanced dressage riders (14). Professional coaches often observe that riders prioritise improving their horse over addressing their own physical limitations, leading to persistent unilateral loading. Such expert opinions highlight a need for greater awareness and targeted physical training to correct inherent asymmetries, improve balance and enhance communication with the horse.

Overall, the literature portrays rider asymmetry as a multifactorial phenomenon with measurable effects on performance and welfare. The deviations in load and movement patterns repeat with every stride, potentially leading to chronic strain on the horse's musculoskeletal system. Evidence-based interventions—such as core-strengthening, physiotherapy, proprioceptive training and equipment optimisation—have shown promise, but the evidence base remains sparse and heterogeneous. Long-term studies are needed to evaluate whether corrective training programmes produce sustained improvements in both riders and horses.

### 2.5 Rider fitness, health, and training practices

Five studies (see Supplementary Table S1, studies 7 and 12–17) explored riders' fitness, health and training practices. Orthopedic issues were a central focus: a case–control study found that lower back pain is prevalent among elite riders, yet imaging did not reveal greater disc degeneration compared with non-riders, suggesting multifactorial origins (33). Surveys reported that up to 68% of riders experience chronic or recurrent back pain, often linked to poor core strength and inadequate warm-up routines (34). Similar findings have been reported in equestrian populations and trunk-focused intervention studies, highlighting the role of core stability in reducing low back pain and improving postural control (48, 49). Neuromuscular analyses showed that advanced riders exhibit greater core engagement and intermuscular coordination than novices, indicating that skill level and conditioning are intertwined (35). Randomised and quasi-experimental interventions demonstrated that horseback riding exercise improves static and dynamic balance more effectively compared with trunk stability exercise, and eight-week core-strength programmes improve postural stability and can reduce perceived exertion (34). Physiological studies measured heart rate, oxygen uptake and lactate accumulation during walk–trot–canter sessions and concluded that riding imposes moderate-to-vigorous cardiovascular demands, therefore endurance training should complement horseback riding (36). Surveys of fitness habits revealed that many riders neglect structured off-horse conditioning and exhibit inadequate energy intake coupled with low quality diet, despite reporting fatigue and poor performance (37, 38).

Studies also explored training practices. Only two empirical investigations explicitly examined warm-up routines. Chatel et al. (39) analysed show-jumping competitions and found that knocking down or refusing fences in the warm-up did not affect competition results. Male riders jumped uprights more frequently than female riders, but this had no performance effect. Riders who had competed earlier in the day spent more time warming up on the flat but attempted fewer jumps. Christensen (40) reported that warm-up intensity influences readiness for jumping but found no significant relationship between warm-up duration and fault accumulation. These findings suggest that while warm-up modes vary by experience and horse age, their impact on performance may be subtle. Reaction time was studied by Williams et al. (41), who found that older riders exhibited slower responses but no clear hand dominance, likely because riders strive for balanced rein contact. Although reaction time declines with age, this may not handicap seasoned equestrians. Extensive experience and exposure to diverse horses enable them to anticipate problems and proactively adjust their aids, reducing the need for rapid reflexive responses.

Overall, the evidence indicates that rider fitness, health and training practices are modifiable factors that influence both performance and horse welfare. Yet many riders do not engage in structured conditioning, emphasising the need for education and targeted interventions.

## 3 Discussion

This review aimed to understand the intricate relationship between rider biomechanics, posture, health, fitness, and horse welfare in equestrian sports. From understanding the impact of stirrup length on impact attenuation to investigating rider asymmetry and its effects on equine locomotion. Our findings showed that researchers have made significant strides in unraveling the complexities of horse-rider dynamics.

Rider Biomechanics and Posture: The reviewed evidence consistently shows that a well-aligned rider position is more than an aesthetic ideal—it directly affects the horse's comfort and performance. Our findings reinforce long-standing equestrian wisdom that a balanced, independent seat is crucial for harmonious riding. Maintaining a neutral pelvis and engaged core was shown to lower saddle pressure and improve horse gait quality (13). These findings align with earlier observations that an unbalanced rider can create pressure points and hinder the horse's movement [as also suggested by (4)]. In practice, riding instructors have always emphasized proper posture, and in recent years, these guidelines have been supported by scientific data. However, many riders still struggle to achieve correct alignment, indicating a need for better training methods or tools (such as real-time feedback devices) to help riders attain an effective position. In the broader context, these results highlight that improving rider biomechanics isn't just about competitive performance—it's also a welfare issue for the horse, echoing the calls for more humane and horse-friendly riding practices (16, 30). Other biomechanical analyses have similarly underscored how even minor deviations in rider position can lead to uneven pressure distribution on the horse's back (16), potentially causing discomfort over time. Thus, our review confirms and extends prior studies by showing that correct rider posture and balance benefit both performance and horse well-being, validating the traditional emphasis on the “classical seat” with scientific evidence (42).

Considering rider asymmetry, nearly all riders have some degree of asymmetry, and our review found that such imbalances can have measurable effects on equine locomotion and welfare (27). This is a concern that has been noted in the equestrian community for decades—for instance, experienced trainers often observe that both horse and rider tend to be “left- or right-handed” and require exercises to even out their asymmetries (26). Earlier studies identified inherent asymmetries in riders' spines and habitual riding patterns that reinforce them (10, 25). Researchers found that riders are frequently unaware of their own crookedness (22), even though objective measurements (e.g., pressure mats, motion analysis) show clear left-right differences in weight distribution and posture. This disparity between perceived and actual alignment means riders might unknowingly be causing uneven loading on the horse (32, 43). Our discussion also connects to older research on laterality. Symes and Ellis (10) noted that horses themselves have natural sidedness, which can in turn affect the rider's position. Such insights place our findings in a broader context considering both human and equine asymmetries interact, making it challenging to achieve perfect symmetry. Practically, this suggests that riders need regular assessments, like physiotherapy evaluations or technological feedback to identify and correct asymmetry. It also indicates that coaching should prioritize symmetry from early on, as experienced years of riding with slight imbalances might lead to chronic back pain or performance issues, as Hobbs et al. (14) observed in elite dressage riders. In summary, our review's asymmetry findings echo earlier studies in acknowledging that asymmetry has multiple causes—from handedness and limb dominance (23) to past injuries—and underline the importance of targeted exercises and perhaps cross-training to mitigate these effects. Addressing rider asymmetry is not only vital for the rider's health but also for the horse development and comfort.

Lastly, Rider Fitness, Health, and Training Practices: The review revealed that a rider's physical fitness and health habits significantly influence both their performance and their horse's welfare (44). This facet has often been underappreciated historically. Traditionally, the focus in equestrian sport was on the horse's fitness, while the rider was sometimes whimsically said to “just sit there”. However, sports science—even as early as the 1980s—began to challenge this notion. For instance, Westerling (45) found that experienced riders working at trot and canter utilized at least 60% of their maximal aerobic capacity, indicating that riding can be a moderately intense workout for the human athlete (36). Our review of recent studies similarly showed that riding imposes moderate-to-vigorous cardiovascular demands on the rider (46), and that many riders report fatigue and even chronic back pain linked to inadequate conditioning (37, 47). These scientific findings mirror practical experience: riders who don't do any off-horse exercise often struggle with core stability and endurance in the saddle. Moreover, they found that structured fitness programs (e.g., core strengthening regimes, aerobic training) can significantly improve riders' balance and reduce their perceived exertion (13). This supports what some forward-thinking coaches have advocated in the last decade—treating riders as true athletes who require cross-training, nutrition planning, and recovery protocols similar to athletes in other sports. Unfortunately, surveys indicate that a large portion of riders neglect regular fitness training and may have suboptimal diets (38). The discussion puts this into a wider context by noting that the equestrian world has started to change: national federations and high-performance programs are increasingly providing guidelines for rider fitness, and research like ours gives credence to those initiatives. In practical terms, the current evidence base, though limited, suggests that riders can enhance their own performance and their horses' well-being through better fitness habits. This area would benefit from more outreach and education—for example, translating research findings into easy-to-follow conditioning programs for amateur riders. It's clear that the horse-rider partnership performs best when both members are fit and pain-free, reinforcing the modern perspective that the rider's body needs as much training and care as the horse's.

### 3.1 Future directions

Building on our findings, future research should aim to fill the gaps identified and employ more robust designs to test interventions. A key recommendation is to undertake longitudinal and experimental studies to evaluate whether addressing rider-specific factors can produce measurable improvements in both rider and horse outcomes. Researchers could design intervention trials where one group of riders undergoes a tailored conditioning program (focused on core strength, symmetry exercises, or aerobic fitness) while a control group maintains their usual routine, tracking the effects on riding performance and horse gait or behavior over time. Similarly, biofeedback and training tools could be tested—such as using real-time pressure sensors or motion sensors to alert riders to asymmetry and seeing if this feedback leads to corrections that benefit the horse. The integration of modern wearable technology offers an especially exciting avenue for future studies. Devices like inertial measurement units (IMUs), pressure mats in saddles, electromyography (EMG) sensors, and global positioning system (GPS) trackers can now capture fine-scale data on how rider and horse move together (43). By using these tools in both training and competition settings, researchers can objectively quantify aspects of the horse–rider interaction that were previously hard to measure. For instance, how different rider physiques or saddle fits affect pressure distribution on the horse's back, or how fatigue over a long ride alters the rider's position and the horse's way of going. Combining such objective measurements with traditional performance metrics and even subjective assessments (like judges' scores or rider/horse stress levels) will help build a more complete picture of effective riding.

Additionally, future studies should not neglect the psychological and cognitive dimensions of rider performance. Factors such as the rider's anxiety, confidence, focus, and the quality of the rider–horse relationship can all influence outcomes and might interact with physical factors (41). Including assessments of rider psychology or decision-making, alongside physical training, could yield insights into how mental and physical preparation together determine success. Finally, translating research into practice will be crucial. As evidence accumulates, it should be used to develop evidence-based guidelines for riders, coaches, and equine veterinarians or physiotherapists. This might include creating standardized fitness assessment protocols for riders (similar to fitness tests used in other sports), best-practice recommendations for addressing asymmetry (perhaps regular screenings or exercise prescriptions), and educational programs to raise awareness about the importance of rider fitness and biomechanics for horse welfare. By pursuing these research directions, the equestrian field can continue to evolve, leveraging scientific advances to enhance the well-being of horses and riders alike and to promote excellence in this unique multi-species sport.

## 4 Conclusions

In conclusion, equestrian sport is unique because success and welfare depend on the harmonious partnership of two athletes (horse and rider). Our narrative review demonstrates that rider-centered factors—biomechanics, asymmetry, and fitness—are critical determinants of this harmony. Proper posture and balanced weight distribution in the saddle lead to more even saddle pressures and better horse movement, whereas uncorrected rider asymmetry or poor fitness can disrupt the horse's gait and potentially compromise welfare. Encouragingly, riders who engage in structured off-horse training (such as strength conditioning and aerobic exercise) tend to exhibit better balance, coordination, and endurance; yet a significant portion of equestrians still neglect these fitness practices. Taken together, the evidence suggests that a holistic approach is essential: combining biomechanical assessments and corrections (to address posture and asymmetry) with tailored fitness and conditioning programs for riders. Such an approach will not only improve rider performance and reduce injury risk but also uphold our ethical responsibility to protect horse welfare. By focusing on the rider as an athlete, equestrian sports can advance into a new era where training and research innovations benefit both members of the horse–rider team. Addressing these rider-centered factors will not only improve performance but also fulfil the ethical commitment to promote horse welfare in a unique multi-species Olympic sport. Finally, as most available studies are small-scale and cross-sectional, more longitudinal research is needed to validate rider-focused interventions and guide evidence-based best practices in equestrian sports.

## References

[1] Note: The following list includes both the 17 empirical studies summarised in Table 1 and additional sources cited in the narrative.

[2] American Horse Council Foundation. The economic impact of the horse industry in the United States. American Horse Council (2017).

[3] Fédération Equestre Internationale. Global equestrian statistics report. Fédération Equestre Internationale. FEI (2021).

[4] International Olympic Committee. Olympic Games sports statistics report. International Olympic Committee. IOC (2020).

[5] Peham C Kotschwar AB Borkenhagen B Kuhnke S Molsner J Baltacis A. A comparison of forces acting on the horse’s back and the stability of the rider’s seat in different positions at the trot. Vet J. (2010) 184(1):56–9. 10.1016/j.tvjl.2009.04.007 19428275

[6] FEI Code of Conduct for the Welfare of the Horse. FEI Code of Conduct for the Welfare of the Horse (2024). Available online at: https://inside.fei.org/sites/default/files/FEI%20Code%20of%20Conduct%20for%20the%20Welfare%20of%20the%20Horse.pdf (Accessed November 4, 2024).

[7] Williams J Tabor G. Rider impacts on equitation. Appl Anim Behav Sci. (2017) 190:28–42. 10.1016/j.applanim.2017.02.019

[8] Licka T Kapaun M Peham C. Influence of rider on lameness in trotting horses. Equine Vet J. (2004) 36(8):734–6. 10.2746/0425164044848028 15656506

[9] de Cocq P Clayton H Terada K Muller M Van Leeuwen JL. Usability of normal force distribution measurements to evaluate asymmetrical loading of the back of the horse and different rider positions. Vet J. (2009) 181:266–73. 10.1016/j.tvjl.2008.03.002 18502669

[10] de Cocq P Duncker AM Clayton HM Bobbert MF Muller M van Leeuwen JL. Vertical forces on the horse’s back in sitting and rising trot. J Biomech. (2010) 43(4):627–31. 10.1016/j.jbiomech.2009.10.036 19926088

[11] Symes D Ellis R. A preliminary study into rider asymmetry within equitation. Vet J (London, England: 1997). (2009) 181:34–7. 10.1016/j.tvjl.2009.03.016 19375366

[12] Roepstorff L Egenvall A Rhodin M Byström A Johnston C van Weeren PR Kinetics and kinematics of the horse comparing left and right rising trot. Equine Vet J. (2009) 41(3):292–6. 10.2746/042516409X397127 19469238

[13] Sukhera J. Narrative reviews in medical education: key steps for researchers. J Grad Med Educ. (2022) 14(4):418–9. 10.4300/JGME-D-22-00481.1 35991097 PMC9380643

[14] Clayton H Hobbs S. The role of biomechanical analysis of horse and rider in equitation science. Appl Anim Behav Sci. (2017) 190:123–32. 10.1016/j.applanim.2017.02.011

[15] Hobbs SJ Baxter J Broom L Rossell LA Sinclair J Clayton HM. Posture, flexibility and grip strength in horse riders. J Hum Kinet. (2014) 42:113–25. 10.2478/hukin-2014-0066 25414745 PMC4234750

[16] Bye TL Lewis V. Footedness and postural asymmetry in amateur dressage riders, riding in Medium trot on a dressage simulator. J Equine Vet Sci. (2021) 102:103618. 10.1016/j.jevs.2021.103618 34119193

[17] Geser-von Peinen K Wiestner T Bogisch S Roepstorff L van Weeren P Weishaupt M. Relationship between the forces acting on the horse’s back and the movements of rider and horse while walking on a treadmill. Equine Vet J. (2009) 41:285–91. 10.2746/042516409X397136 19469237

[18] Wilkins CA Wheat JS Protheroe L Nankervis K Draper SB. Coordination variability reveals the features of the “independent seat” in competitive dressage riders. Sports Biomech. (2025) 24(2):372–87. 10.1080/14763141.2022.2113118 35993195

[19] Keener MM Critchley ML Layer JS Johnson EC Barrett SF Dai B. The effect of stirrup length on impact attenuation and its association with muscle strength. J Strength Cond Res. (2021) 35(11):3056–62. 10.1519/JSC.0000000000003278 31972822

[20] Stapley ED Stutzman BE Manfredi JM. The effect of stirrup iron style on normal forces and rider position. J Equine Vet Sci. (2020) 94:103203. 10.1016/j.jevs.2020.103203 33077067

[21] Randle H Loy J. First steps to establishing an equestrian morphology: can vitruvian ratios help? Comp Exerc Physiol. (2019) 16:1–12. 10.3920/CEP190041

[22] Bye T Lewis V. Saddle and stirrup forces of equestrian riders in sitting trot, rising trot, and trot without stirrups on a riding simulator. Comp Exerc Physiol. (2019) 16:1–12. 10.3920/CEP190031

[23] MacKechnie-Guire R MacKechnie-Guire E Fairfax V Fisher M Hargreaves S Pfau T. The effect that induced rider asymmetry has on equine locomotion and the range of motion of the thoracolumbar spine when ridden in rising trot. J Equine Vet Sci. (2020) 88:102946. 10.1016/j.jevs.2020.102946 32303298

[24] Bagesteiro L Sainburg R. Handedness: dominant arm advantages in control of limb dynamics. J Neurophysiol. (2002) 88:2408–21. 10.1152/jn.00901.2001 12424282 PMC10709816

[25] Kellis E Katis A. Biomechanical characteristics and determinants of instep soccer kick. J Sports Sci Med. (2007) 6(2):154–65. 24149324 PMC3786235

[26] Bussey M. Does the demand for asymmetric functional lower body postures in lateral sports relate to structural asymmetry of the pelvis? J Sci Med Sport/Sports Med Aust. (2009) 13:360–4. 10.1016/j.jsams.2009.02.010 19596607

[27] Eisersiö M Rhodin M Roepstorff L Egenvall A. Rein tension in 8 professional riders during regular training sessions. J Vet Behav. (2015) 10(5):419–26. 10.1016/j.jveb.2015.05.004

[28] Byström A Clayton H Hernlund E Roepstorff L Rhodin M Braganca F Asymmetries of horses walking and trotting on treadmill with and without rider. Equine Vet J. (2020) 53:157–66. 10.1111/evj.13252 32125717

[29] Clayton H MacKechnie-Guire R Hobbs S. Riders’ effects on horses—biomechanical principles with examples from the literature. Animals (Basel). (2023) 13:3854. 10.3390/ani13243854 38136891 PMC10741103

[30] Clayton H MacKechnie-Guire R Byström A Le Jeune S Egenvall A. Guidelines for the measurement of rein tension in equestrian sport. Animals (Basel). (2021) 11(10):2875. 10.3390/ani11102875 34679895 PMC8532849

[31] Hogg RC Hodgins GA. Symbiosis or sporting tool? Competition and the horse-rider relationship in elite equestrian sports. Animals (Basel). (2021) 11(5):1352. 10.3390/ani11051352 34068606 PMC8151029

[32] Elbrønd VS Schultz RM. Deep myofascial kinetic lines in horses, comparative dissection studies derived from humans. Open J Vet Med. (2021) 11(01):14–40. 10.4236/ojvm.2021.111002

[33] Gunst S Dittmann MT Arpagaus S Roepstorff C Latif SN Klaassen B Influence of functional rider and horse asymmetries on saddle force distribution during stance and in sitting trot. J Equine Vet Sci. (2019) 78:20–8. 10.1016/j.jevs.2019.03.215 31203980

[34] Kraft CN Pennekamp PH Becker U Young M Diedrich O Lüring C Magnetic resonance imaging findings of the lumbar spine in elite horseback riders: correlations with back pain, body mass Index, trunk/leg-length coefficient, and riding discipline. Am J Sports Med. (2009) 37(11):2205–13. 10.1177/0363546509336927 19574474

[35] Kim H Lee C-W Lee I-S. Comparison between the effects of horseback riding exercise and trunk stability exercise on the balance of normal adults. J Phys Ther Sci. (2014) 26:1325–7. 10.1589/jpts.26.1325 25276009 PMC4175230

[36] Elmeua M Sarabon N. Muscle modes of the equestrian rider at walk, rising trot and canter. PLoS One. (2020) 15:e0237727. 10.1371/journal.pone.0237727 32810165 PMC7446812

[37] Hyttinen A-M Ahtiainen J Häkkinen K. Oxygen uptake, heart rate and blood lactate levels in female horseback riders during the obstacle test track. Int J Perf Anal Sport. (2020) 20:1–12. 10.1080/24748668.2020.1764747

[38] Bye T Chadwick G. Physical fitness habits and perceptions of equestrian riders. Comp Exerc Physiol. (2018):1–6. 10.3920/CEP180012

[39] Best R Williams J Pearce J. The physiological requirements of and nutritional recommendations for equestrian riders. Nutrients. (2023) 15:4977. 10.3390/nu15234977 38068833 PMC10708571

[40] Chatel MM Tabor G Williams JR Williams J. An evaluation of factors affecting show jumping warm-up on subsequent show jumping performance in 1.30 m class. Comp Exerc Physiol. (2020) 17:1–14. 10.3920/CEP200026

[41] Christensen J Munk R Hawson L Palme R Larsen T Egenvall A Rider effects on horses’ conflict behaviour, rein tension, physiological measures and rideability scores. Appl Anim Behav Sci. (2020) 234:105184. 10.1016/j.applanim.2020.105184

[42] Williams JM Douglas J Davies E Bloom F Castejon-Riber C. Performance demands in the endurance rider. Comp Exerc Physiol. (2021) 17:199–218. 10.3920/CEP200033

[43] Wilkins C. Dynamic technique analysis of the female equestrian rider (Thesis). Celeste Wilkins, Gloucester, England (2021). Available online at: https://search.ebscohost.com/login.aspx?direct=true&db=edsble&AN=edsble.833909<=hu&site=eds-live

[44] Baragli P Alessi A Pagliai M Felici M Ogi A Hawson L Rider variables affecting the stirrup directional force asymmetry during simulated riding trot. Animals (Basel). (2022) 12:3364. 10.3390/ani12233364 36496885 PMC9737979

[45] O’Reilly C Zoller J Sigler D Vogelsang M Sawyer J Fluckey J. Rider energy expenditure during high intensity horse activity. J Equine Vet Sci. (2021) 102:103463. 10.1016/j.jevs.2021.103463 34119194

[46] Westerling D. A study of physical demands in riding. Eur J Appl Physiol Occup Physiol. (1983) 50(3):373–82. 10.1007/BF00423243 6683161

[47] Devienne MF Guezennec CY. Energy expenditure of horse riding. Eur J Appl Physiol. (2000) 82(5–6):499–503. 10.1007/s004210000207 10985607

[48] Ferrante M Bonetti F Quattrini F Mezzetti M Demarie S. Low back pain and associated factors among Italian equestrian athletes: a cross-sectional study. Muscle Ligaments Tendons J. (2021) 11:344. 10.32098/mltj.02.2021.19

[49] Carpes F Reinehr F Mota C. Effects of a program for trunk strength and stability on pain, low back and pelvis kinematics, and body balance: a pilot study. J Bodyw Mov Ther. (2008) 12:22–30. 10.1016/j.jbmt.2007.05.001 19083652

[50] Duarte C Raimundo A Sousa JP Fernandes O Santos R. Prevalence of lower back pain and risk factors in equestrians: a systematic review. Sports. (2024) 12:355. 10.3390/sports12120355 39728895 PMC11679230

