# Supplementary material for: A narrative review of factors influencing rider performance and horse welfare in equestrian activities
Source: Front Sports Act Living. 2026 Jan 22;7:1744918. doi: 10.3389/fspor.2025.1744918 (PMC12872785; doi:10.3389/fspor.2025.1744918)
Supplement: Supplementary file 1 [file Table1.docx]

**Supplementary Table 1. Summary of Empirical Studies Included in the Review**

The table below presents the seventeen empirical studies that met the inclusion criteria for this narrative review. Studies were selected based on their focus on riders rather than horses, the presence of quantitative data, and relevance to at least one of the three research questions (rider biomechanics and posture, rider asymmetry, or rider fitness and training). Each study is summarised with regard to its design, sample, focus and key findings.

| No. | Author & year | Country | Design | Sample & discipline | Focus area | Key findings |
| --- | --- | --- | --- | --- | --- | --- |
| 1 | Clayton & Hobbs (2017) | UK | Cross-sectional motion analysis | 20 dressage riders; intermediate to elite | Biomechanics (RQ1) | Proper pelvis tilt and trunk control associated with improved seat stability and reduced saddle pressure. |
| 2 | Hobbs et al. (2014) | UK | Observational kinematic study | 12 experienced riders | Biomechanics (RQ1) | Postural asymmetries, reduced lateral bending range of motion and grip strength differences were observed in experienced riders, suggesting long-term adaptations related to riding experience. |
| 3 | Geser-von Peinen et al. (2009) | Switzerland | Force-plate study | 8 riders on horse on treadmill | Biomechanics (RQ1) | Symmetrical weight distribution reduced uneven saddle forces; proper stirrup length improved balance. |
| 4 | MacKechnie-Guire et al. (2020) | UK | Experimental biomechanical study | 10–12 horse–rider pairs | Biomechanics & asymmetry (RQ1 & RQ2) | Induced rider asymmetry significantly altered equine thoracolumbar range of motion. Asymmetrical loading resulted in measurable changes in horse movement patterns. |
| 5 | de Cocq et al. (2009) | Netherlands | Cross-over experiment | 7 riders on mechanical horse | Biomechanics (RQ1) | Seat positioned near horse’s centre of mass reduced saddle pressure and improved comfort. |
| 6 | Christensen et al. (2020) | Denmark | Cross-over experiment | 20 horse–rider pairs | Biomechanics (RQ1) | Increased rider weight resulted in higher rein tension and increased conflict-related behaviours in horses. Physiological stress markers increased with heavier rider load. |
| 7 | Elmeua González & Šarabon (2020) | Slovenia | Observational neuromuscular study | 6 novice & 9 professional riders | Biomechanics & fitness (RQ1 & RQ3) | Advanced riders displayed higher core muscle engagement and better intermuscular coordination; novice riders used inefficient muscle patterns. |
| 8 | Gunst et al. (2019) | Switzerland | Observational biomechanical study | Horse–rider pairs | Asymmetry (RQ2) | Both rider and horse asymmetries significantly affected saddle force distribution. The interaction between rider and horse asymmetry contributed to uneven pressure distribution on the horse’s back.. |
| 9 | Symes & Ellis (2009) | Germany | Cross-sectional motion analysis | 17 riders | Asymmetry (RQ2) | Shoulder displacement and pelvic tilt differed between left and right sides; recommended targeted stretching and physiotherapy to improve symmetry. |
| 10 | Baragli et al. (2022) | Italy | Force measurement study | 16 amateur riders | Asymmetry (RQ2) | Left–right differences in stirrup force were associated with riding style and previous injuries; asymmetries were consistently observed during simulated trot. |
| 11 | Bye & Lewis (2021) | UK | Simulator-based observational study | Amateur dressage riders | Asymmetry (RQ2) | Riders compensate for asymmetry using stirrups; pelvic obliquity and shoulder tilt observed. |
| 12 | Wilkins et al. (2021) | UK | Biomechanical coordination analysis | Competitive dressage riders | Biomechanics & fitness (RQ1 & RQ3) | Coordination variability affects ability to maintain independent seat and stability. |
| 13 | Keener et al. (2021) | USA | Experimental impact attenuation study | Equestrian riders | Biomechanics (RQ1) | Shortened stirrups reduce lumbar impacts; core strength aids impact attenuation. |
| 14 | Kim et al. (2014) | Korea | Randomized controlled trial | Normal adults | Fitness (RQ3) | Horseback riding improves balance more effectively than trunk stability exercise. |
| 15 | Ferrante et al. (2021) | Italy | Online survey | 886 riders | Fitness (RQ3) | High prevalence of lifetime and 1-year low back pain among competitive riders; body weight and discipline associated with LBP risk. |
| 16 | Bye & Chadwick (2018) | UK | Online survey | 299 riders across disciplines | Fitness (RQ3) | Most riders lacked structured fitness routines; those engaging in cross-training reported better performance and reduced fatigue. |
| 17 | Best et. al (2023) | - | Nutritional survey | 50 competitive riders | Fitness (RQ3) | Riders showed inadequate energy intake and poor macronutrient balance; nutritional guidance recommended to support performance. |
